# What Has Changed in the Management of Uterine Serous Carcinomas? Two Decades of Experience

**DOI:** 10.3390/curroncol28060410

**Published:** 2021-11-20

**Authors:** Michalis Liontos, Anna Svarna, Charalampos Theofanakis, Oraianthi Fiste, Angeliki Andrikopoulou, Maria Kaparelou, Konstantinos Koutsoukos, Nikolaos Thomakos, Dimitrios Haidopoulos, Alexandros Rodolakis, Meletios Athanasios Dimopoulos, Flora Zagouri

**Affiliations:** 1 Department of Clinical Therapeutics, Alexandra Hospital, National and Kapodistrian University of Athens, 11528 Athens, Greece; anna.svarna@hotmail.com (A.S.); ofiste@med.uoa.gr (O.F.); aggandrikop@med.uoa.gr (A.A.); mkaparelou@yahoo.com (M.K.); koutsoukos.k@gmail.com (K.K.); mdimop@med.uoa.gr (M.A.D.); florazagouri@yahoo.co.uk (F.Z.); 2 1st Department of Obstetrics and Gynecology, Alexandra Hospital, National and Kapodistrian University of Athens, 11528 Athens, Greece; ctheofan@med.uoa.gr (C.T.); nthomakos@med.uoa.gr (N.T.); dchaidop@med.uoa.gr (D.H.); arodolak@med.uoa.gr (A.R.)

**Keywords:** cancer, endometrial, p53 abnormal expression, survival

## Abstract

Uterine serous carcinoma accounts for 3–10% of endometrial cancers, but it is the most lethal histopathological subtype. The molecular characterization of endometrial carcinomas has allowed novel therapeutic approaches for these patients. We undertook a retrospective analysis of patients with uterine serous carcinomas treated in our hospital within the last two decades to identify possible changes in their management. The patients and their characteristics were evenly distributed across the two decades. Treatment modalities did not change significantly throughout this period. After adjuvant treatment, patients’ median disease-free survival was 42.07 months (95% CI: 20.28–63.85), and it did not differ significantly between the two decades (*p* = 0.059). The median overall survival was 47.51 months (95% Cl: 32.18–62.83), and it significantly favored the first decade’s patients (*p* = 0.024). In patients with de novo metastatic or recurrent disease, median progression-free survival was 7.8 months (95% Cl: 5.81–9.93), whereas both the median progression-free survival and the median overall survival of these patients did not show any significant improvement during the examined time period. Overall, the results of our study explore the minor changes in respect of uterine serous carcinoma’s treatment over the last two decades, which are reflected in the survival outcomes of these patients and consequently underline the critical need for therapeutic advances in the near future.

## 1. Introduction

Endometrial cancer is the most frequent gynecological cancer in developed countries. According to the American Cancer Society (ACS), more than 65,000 women will be diagnosed and about 12,500 will eventually die from this disease in 2021. Women aged 60 to 70 are primarily in peril, with 95–98% of tumors affecting women above the age of 40 [1]. The most common histological type is endometrioid at about 85%, while the serous type of uterine carcinomas (USC) comes far second, at a rate of 3–10% of all cases [2]. Despite the small percentage of women carrying this histological subtype, a disproportionate number of them will succumb compared to the endometrioid type group. More specifically, approximately 40% of uterine cancer-related deaths are attributed to the serous variant [3].

USC’s aggressive biological behavior could elucidate its distinct clinical course; it is usually diagnosed at a more advanced stage, and it is associated more frequently with extra-pelvic recurrence [4,5]. Consequently, USC’s 5-year survival rates do not exceed 45% [6,7] and even in the best performing stage I patients, the 5-year survival rate is about 70%, which is considerably worse than in patients with the endometrioid type [8].

The widely used Bokhman model categorizes endometrial cancer into two pathogenetic types according to its clinical characteristics as well as its metabolic and endocrine phenotype: type I is the prototypical endometrioid endometrial cancer, and type II is the prototypical serous endometrial carcinoma [9]. However, the Cancer Genome Atlas (TCGA) comprehensive molecular profiling of endometrial carcinomas and the new FIGO classification have divided endometrial cancers into four prognostic subgroups: ultramutated (POLEmut), hypermutated/mismatch repair deficient (MMRd), copy-number high driven by oncogene TP53 (p53abn), and copy-number low without a specific driver mutation (NSMP). USC is classified into the p53abn group, which is characterized by low mutational burden, genomic instability, frequent TP53 mutations, and thus unfavorable outcomes [10,11]. Indeed, the abnormal immunohistochemical expression of p53 represents an adverse prognostic feature, rendering USCs as high-risk carcinomas that should be treated with adjuvant chemotherapy and radiotherapy as per the PORTEC-3 study [12,13].

Molecular testing is now recommended for all newly diagnosed uterine carcinomas, given that molecular classification of the uterine carcinomas is incorporated in the recently published 2020 ESGO/ESTRO/ESP guidelines as a determinant of therapeutic decisions [14]. Molecular testing also allows targeted agents to be included as treatment options in recurrent endometrial cancer following the updated guidelines [14]. More specifically, trastuzumab has shown efficacy for Her-2 amplified patients [15], whilst the pembrolizumab/lenvatinib combination demonstrated promising results in the recurrent setting [16]. Hence, several novel agents (anti-PD1 and anti-PD-L1 molecules, PARP inhibitors, selective inhibitors of nuclear export, etc.) are currently under investigation in endometrial cancer clinical trials, which are often based on novel molecular taxonomy trial designs [17,18,19,20,21,22,23,24].

Under this perspective, we aim to examine the clinical course and management of patients diagnosed with USC in our institution over a 20-year period. The objective of this study is to further appraise patients’ clinical characteristics along with survival outcomes and compare the collected data in these two time periods (1999–2009, 2010–2019) in order to unveil potential differences concerning treatment and mortality throughout the years.

## 2. Materials and Methods

### 2.1. Patients

Patients with histologically confirmed USC treated in our Oncology Unit between 1999 and 2019 were retrospectively identified for analysis. Patients with a histopathological diagnosis of pure serous endometrial carcinoma or mixed serous carcinoma (defined as tumors with at least a 10% serous component) were included. All patients had provided written informed consent for the use of their medical records for research purposes. The study was approved by our Institutional Review Board and was conducted according to the Declaration of Helsinki.

Clinicopathological, treatment-related, and survival data were collected through a single-institution database. More precisely, demographic data, including patients’ date of birth, age at diagnosis, and date of first disease progression and/or death were documented. The extent of resection, type of radiotherapy (e.g., External Beam Radiotherapy (EBRT), brachytherapy, or a combination of the two), and data regarding chemotherapy regimens were also recorded. Tumor staging was performed in accordance with the International Federation of Gynecology and Obstetrics (FIGO) classification system for uterine adenocarcinomas of 2014. The patients’ performance status was measured according to ECOG Scale Performance Status [25]. Disease progression was defined, according to the Response Evaluation Criteria in Solid Tumors (RECIST), as a new metastatic lesion and/or an ≥20% increase in the sum of diameters of target lesions and/or clinical deterioration. Death was not assessed as being cancer-related or non-cancer-related.

### 2.2. Statistical Analysis

Descriptive statistics were used to provide information about the variable parameters of the patients. Continuous variables were summarized with the use of descriptive statistical measures (median and percentiles (25th, 75th)), whereas categorical variables were displayed as frequency tables (*N*, %). Overall survival (OS), disease-free survival (DFS), and progression-free survival (PFS) were calculated as part of the survival analysis. OS was calculated as the interval between the date of initial diagnosis and the date of death from any cause, or the date of last alive contact, or lost to follow-up. DFS was defined as the time from primary treatment (surgery) until disease recurrence or death from any cause for patients with initial stage IA–IIIc. PFS was defined as the length of time from diagnosis of advanced (stage IV) or recurrent disease until further disease progression or death from any cause.

The Kaplan–Meier survival analysis was generated to estimate both the probability of disease recurrence and death over time. The log-rank test was used to compare the prognostic value of categorical variables on survival curves. Univariable and multivariable Cox proportional hazard models assessed the Hazard Ratios (HR) and the 95% Confidence Intervals (CI). All tests were two-sided, while statistical significance was defined as *p*-value < 0.005. All statistical analyses were performed using the SPSS software.

## 3. Results

### 3.1. Patients’ Characteristics

Between January 1999 and December 2019, 121 women with a USC diagnosis were identified, and their clinical data were analyzed. Demographic and clinicopathological characteristics are presented in Table 1. For a limited number of non-operated patients, the stage could not be appropriately defined, and their stage data were classified as missing. Briefly, 66 (54.5%) had pure serous carcinoma, whereas 55 had mixed histology, with a median age of 66.49 years (25th and 75th percentiles 60.6–72.6 years). Almost all patients included (115/121) in the study had received prior surgery. Lymphadenectomy was conducted in 63 cases (54.8%) and omentectomy was conducted in 83 cases (72.2%), with the exception of two patients whose surgery records were unavailable. Overall, 27.3% (33/121) of the women were diagnosed with stage IA USC, while 23.1% (28/121) were presented with de novo metastatic disease. In total, 91 women were eligible for adjuvant therapy: 50 women in the 1st time period (1999–2009) and 41 in the 2nd decade (2010–2019). Of the 87 women who received adjuvant treatment, 47 (54%) received chemotherapy alone, 38 (43.6%) received chemotherapy and radiation therapy, whereas 2 patients (2.3%) received only radiotherapy. Treatment modalities were evenly distributed over the 2 decades (*p =* 0.15). No statistically significant differences were found between the baseline characteristics of patients diagnosed in the 1st decade (1999–2009) and the 2nd decade (2010–2019) of our study (Table 1).

### 3.2. Adjuvant Treatment

**Figure 1:**
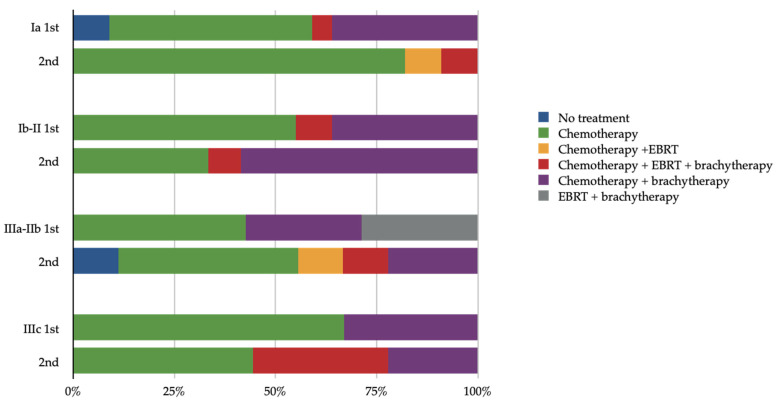
Adjuvant treatment in relation to stage and decade of treatment (1st decade: 1999–2009, 2nd decade: 2009–2019, EBRT: external beam radiotherapy).

Post-surgical treatment, 91 had been evaluated as candidates for adjuvant treatment; yet, four patients did not receive any adjuvant therapy. More specifically, the women undergoing combination treatment (chemotherapy and radiation) received mainly brachytherapy as their radiation regimen (28/91, 30.7%) while eight women (8/91, 8.8%) received both external beam radiotherapy (EBRT) and brachytherapy, and two women (2/91, 2.2%) received EBRT only. Those treated with only radiotherapy underwent a combination of EBRT and brachytherapy (2/91, 2.3%). Regarding chemotherapy regimens, the most frequently used was the combination of carboplatin and paclitaxel according to the guidelines that were in place at the time. The types of adjuvant treatment per stage and decade are shown in Figure 1.

**Figure 2:**
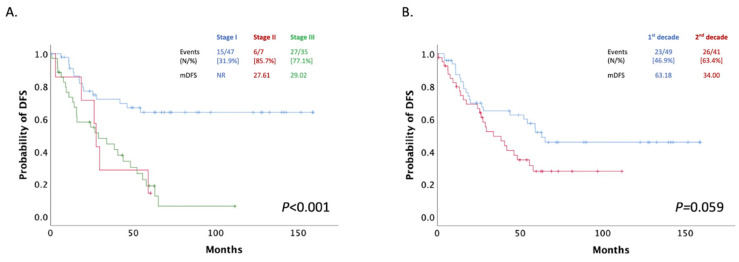
(**A**) Patients mDFS stratified by stage; (**B**) Patients mDFS after adjuvant treatment stratified by decade of treatment (1st decade: 1999–2009, 2nd decade: 2009–2019).

The patients’ median disease-free survival (mDFS) was 42.07 months (95% CI: 20.28–63.85) after the initiation of adjuvant therapy and was significantly associated with stage. In detail, the mDFS for stage I was not reached in our study, while in stage III, mDFS was 29.02 months (95% CI: 5.22–52.83) (Figure 2A). A total of 40 women did not progress during our research’s follow-up period. Moreover, no significant difference has been reported for DFS among the two decade groups (1999–2009 and 2010–2019; *p =* 0.059) (Figure 2B).

### 3.3. First-Line Treatment

A total of 61 patients with recurrent or de novo metastatic disease received first-line treatment and were proportionately distributed between the two decade groups (30 patients in the 1st and 31 in the 2nd decade).

**Figure 3:**
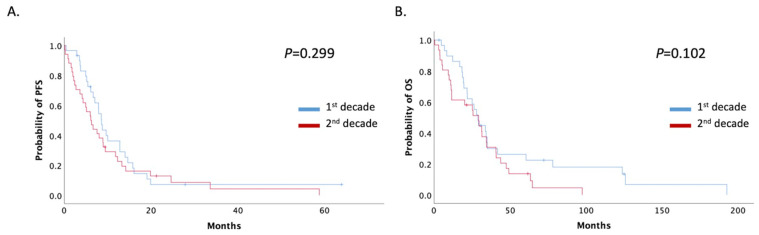
(**A**) Patients with recurrent or de novo metastatic disease mPFS stratified by decade of treatment (1st decade: 1999–2009, 2nd decade: 2009–2019); (**B**) Patients with recurrent or de novo metastatic disease mOS stratified by decade of treatment (1st decade: 1999–2009, 2nd decade: 2009–2019).

The patients’ median progression-free survival (mPFS) in this setting was 7.8 months (95% Cl: 5.81–9.93) and did not differ significantly between the two time-period groups, even when patients were stratified by decade and stage. mPFS was 8.56 (95% CI: 7.44–9.68) and 6.10 (95% CI: 3.94–8.26) months in the 1st and 2nd decade respectively (*p =* 0.299) (Figure 3A). Median overall survival (mOS) also did not change significantly between the two decades (28.82 months; 95% CI: 23.64–34.01 vs. 29.34 months; 95% CI: 17.06–41.62; *p =* 0.102) (Figure 3B).

### 3.4. Overall Survival

**Figure 4:**
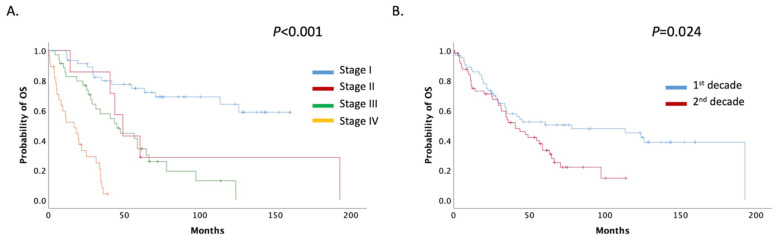
(**A**) Patients mOS stratified by stage; (**B**) Patients mOS stratified by decade (1st decade: 1999–2009, 2nd decade: 2009–2019).

For all patients with USC treated at our institute during the decades 1999–2009 and 2010–2019, mOS was 47.51 months (95% CI: 32.18–62.83). Patients were stratified by stage; mOS was not reached in stage I. However, for stages II, III, and IV, mOS was 49.18, 45.25, and 16.85 months, respectively (Figure 4A).

mOS was significantly decreased among the two decade groups; for the period 1999–2009, mOS was 78.10 (95% CI: 0.00–163.885) months, whereas for the period 2010–2019, it was 40.75 (95% CI: 25.96–55.54) months (*p =* 0.024) (Figure 4B). No overall difference in the survival rate was noted between mixed and pure USCs (60.52 months, 95%CI 34.46–86.57 vs. 40.75 months, 95% CI 26.59–54.90 months, *p =* 0.130) (Supplemental Figure S1).

### 3.5. Multivariate Analysis

A multivariate analysis was conducted to delineate independent predictors of unfavorable survival outcomes further. Patients’ characteristics such as age, histology, and stage (I (IA, IB), II, III (IIIA, IIIB)), type of surgery (lymphadenectomy, omentectomy), type of adjuvant treatment, and the period of diagnosis (1st or 2nd decade) were included. Stage was the sole independent prognostic factor in the multivariate analysis (Table 2).

## 4. Discussion

In this study, we showed that patients and their characteristics were evenly distributed over the two decades, and treatment modalities did not change significantly throughout this period. Survival data concerning these two decades showed that patients’ mDFS after adjuvant treatment and patients’ mPFS and mOS in de novo metastatic or recurrent disease did not differ significantly between the two decades. However, the patients’ mOS significantly favored patients in the first decade.

Taken as a whole, several interesting conclusions were drawn from this retrospective study. At first, we elucidate the clinical characteristics of USC patients in Greece at diagnosis. These observations are consistent with the epidemiological evidence of endometrial cancer patients. At presentation, the median age is 63 years old, yet USC is more frequently reported in patients 5 to 10 years older, with a study having a mean age of diagnosis of 68.7 years [26]. Moreover, 50–70% of these patients are diagnosed with advanced-stage disease [3,27,28,29,30]. Furthermore, in our series, 45.5% of women had mixed type endometrial cancer, which is another well-documented characteristic of uterine papillary serous carcinoma [31], as it has been described that mixed carcinomas are possibly serous carcinomas displaying endometrioid mimicry [32]. On multivariable analysis, mixed type was not found to be significantly associated with survival, and patients with mixed and pure USC had a comparable survival rate, which is a result that has been debated in the bibliography with contradicting results from varying studies [13,33,34].

Moreover, with respect to the real-world management of USC patients in Greece, the surgical interventions included omentectomy and pelvic lymphadenectomy in the majority of the operable cases, which were aligned with the international guidelines [35]. It should be noted though that in approximately 40% of cases, no lymphadenectomy was performed in our series. This percentage is analogous to those reported in various clinical series [36,37,38]. Yet, lymphadenectomy is required for the optimal staging of uterine carcinomas and is correlated with survival benefit in several studies [36,37]. However, these results have been criticized, and more studies are now in progress to further evaluate the clinical significance of lymphadenectomy in endometrial cancer [39].

In our study, chemotherapy was the most common therapeutic modality used as adjuvant treatment. Unfortunately, our study’s limited number of cases did not allow further analysis of the varying treatments used by decade and/or stage. Nevertheless, it is evident that chemotherapy without radiation therapy (EBRT and/or brachytherapy) was initiated in the majority of cases with either early-stage (I/II) or locally advanced disease. Recent data from PORTEC-3 study proved the survival benefit of chemotherapy addition in uterine serous carcinomas [12,40]. Similar results were obtained from the retrospective study of Nasioudis et al. for early-stage USC [41]. Additionally, our findings accentuate the diversity of treatments and their combination used in the adjuvant setting in this patients’ cohort, which are consistent with previous studies in other countries [42,43] and reflect the corresponding treatment options suggested by international guidelines [35]. Molecular classification of the disease and guidelines that have incorporated clinical and molecular evidence of the disease will lay the foundation for personalized treatment in the near future. Currently, the RAINBO umbrella trial attempts to redefine adjuvant therapy in endometrial carcinomas on the basis of molecular testing. More specifically, in patients with p53abn disease, the study will evaluate the role of PARPi maintenance treatment post chemotherapy and radiotherapy [44].

Survival data concerning patients throughout the two-decades period also align with the epidemiological data of USC patients from other centers, whereas survival rates vary, yet prognosis remains unanimously poor [4,45]. The 5-year survival rates of USC patients were found to fluctuate within about 92–74.5%, 66.7–56.7%, 35.7–34.2%, and 17.3–12% for stages I, II, III, and IV, respectively [5,26,46]. As noted above, USC features aggressive behavior portending a dismal prognosis [7]. In our analysis, the stage-stratified 5-year survival rate of our patients was slightly lower compared to the survival rates of previously published studies, which could be attributed in part to inherent differences of surgical approaches; only 52.1% of our USC cases had undergone lymphadenectomy. Lymphadenectomy is recommended by the updated ESGO/ESTRO/ESP guidelines with sentinel lymph node (SNL) being an alternative for stages I/II [14]. Regarding sentinel lymph node dissection, in a retrospective subgroup analysis of 85 patients with clinical stage I USC, 10% of USC patients with no myoinvasion on final pathology had exhibited tumor cells in the SLN, underlining the importance of SLN mapping with pathologic ultrastaging, when feasible, in guiding adjuvant treatment, irrespective of the degree of myoinvasion [47].

However, over the last two decades, little has changed with respect to treatment algorithms. As mentioned above, serous carcinomas have been considered high risk and until recently were treated with adjuvant radiotherapy and chemotherapy according to their stage [35,45,48,49,50]. This lack of change in clinical practice is also depicted in our data. Therefore, no significant differences were found between the two decades regarding mDFS and mPFS after the initial treatment (adjuvant for early-stage and 1 st-line therapy for de novo metastatic disease, respectively). In multivariate analysis, only the stage was found to be an independent prognostic factor, despite the fact that the limited number of stage II patients included in our cohort may skew the exact additional hazard ratio (HR) of these patients. However, the clear difference between stage I and stages II and III HR is the main evidence of our study that stage is an independent prognostic factor. Interestingly, when comparing the mOS between the two decade periods, patients of the 1st time period (1999–2009) displayed a statistically significant increased mOS. A possible explanation for this intriguing finding could be the extended follow-up period, given that patients diagnosed closely to the study’s cut-off date could not produce mature data with respect to OS.

To the best of our knowledge, the present study is the first to appraise trends in both clinical characteristics and the management of USC patients treated at a reference site for Gynecological Oncology in Greece over a 20-year period. Thus, these data not only could allow for a thorough description of potential changes regarding this cohort of patients but could also serve as a platform to incorporate novel treatment strategies in patients with USC in Greece.

The intensive work to implement molecular testing into routine clinical practice as a risk stratification and prognostic tool for USC is ongoing. Indeed, molecular classification is recommended in the updated ESGO/ESTRO/ESP guidelines, albeit the fact that serous carcinomas belong to the p53-aberrant group; thus, they are considered as high-risk uterine neoplasms [14,51]. Nonetheless, a subset of USC can be categorized into lower-risk subgroups in the presence of advantageous mutations, such as POLE [52]. Molecular testing has also given rise to novel treatment strategies based on tumor-specific mutations. Targeting Her-2 amplification, which has been reported in 10–62% of patients with USC [53,54,55,56], has already exhibited clinical benefit and is incorporated in the 2020 ESGO/ESTRO/ESP guidelines [14,15]. Several molecular signaling pathways, including the PI3K/AKT/mTOR pathway and mechanism of Poly (ADP-Ribose) Polymerase-1 (PARP-1), are under investigation for the development of novel transformative therapies that have the potential to impact patients’ survival [57,58]. Ongoing randomized clinical trials that enroll patients with USC evaluate in the first-line setting, the efficacy and safety of (1) Antiangiogenetic treatment plus chemotherapy [59], (2) PARPi and anti-PD-1/PD-L1 combinations as maintenance treatment after first-line chemotherapy [60], and (3) the Selective inhibitor of Nuclear Export (SINE), Selinexor as maintenance treatment post platinum-based chemotherapy [61].

However, this study has some methodological limitations, since it is a retrospective series of patients. Furthermore, the immature OS data of the 2nd decade period (2010–2019) should also be addressed. However, all patients were treated at the same institution, both the baseline characteristics of patients and treatment modalities were evenly distributed between the two decade groups, whilst follow-up was considered adequate to draw useful conclusions for this cohort of patients.

## 5. Conclusions

To conclude, further research is warranted to enable a better understanding of this aggressive variant of endometrial cancer. By opening up new horizons for a molecular-based, individualized, patient care, there is hope for survival benefit, which is the true drive behind scientific research.

## Figures and Tables

**Table 1:** Patients’ characteristics.

Patient Characteristics	All Patients	1st Decade	2nd Decade	*p*-Value
Age (median, 95% Cl)	66.49 (64.9–67.8)	65.9 (63.8–68.1)	66.7 (64.4–68.9)	0.777
	***n*** **(%)**	***n*** **(%)**	***n*** **(%)**	
**Stage**				0.368
IA	33 (27.3%)	22 (34.4%)	11 (19.3%)	
IB-II	23 (19.0%)	11 (17.2%)	12 (21.1%)	
III	34 (28.1%)	16 (25.0%)	18 (31.6%)	
IV	28 (23.1%)	13 (20.3%)	15 (26.3%)	
Missing	3 (4.7%)	2 (3.1%)	1 (1.8%)	
**Lymph node dissection**				0.129
No	50 (41.3%)	23 (35.9%)	27 (47.4%)	
Yes	63 (52.1%)	38 (59.4%)	25 (43.9%)	
Missing	2 (1.6%)	1 (1.6%)	1 (1.7%)	
Not applicable	6 (5%)	2 (3.1%)	4 (7%)	
**Omentum excision**				0.610
No	30 (24.8%)	15 (23.4%)	15 (26.3%)	
Yes	83 (68.6%)	46 (71.8%)	37 (64.9%)	
Missing	2 (1.6%)	1 (1.6%)	1 (1.7%)	
Not applicable	6 (5%)	2 (3.1%)	4 (7%)	
**Type of adjuvant therapy**				0.745
Chemotherapy	47 (38.8%)	26 (40.6%)	21 (36.8%)	
Radiotherapy	2 (1.7%)	2 (3.1%)	0 (0%)	
Chemotherapy and radiotherapy	38 (31.4%)	19 (29.7%)	19 (33.3%)	
Not applicable	34 (28.1%)	17 (26.6%)	17 (29.8%)	

**Table 2:** Multivariant analysis of patients’ overall survival.

Patients’ Characteristics	HR	95% CI	*p*-Value
**Age**			0.771
<65 years	1		
>65 years	1.132	0.492–2.607	
Histology			0.925
Only serous	1		
Mixed	0.962	0.428–2.162	
**Stage**			0.027
I	1		
II	4.147	0.814–21.125	
III	2.716	1.238–5.959	
**Type of adjuvant**			0.268
Chemotherapy	1		
Chemotherapy plus radiotherapy	0.626	0.273–1.435	
**Lymph node dissection**			0.179
No	1		
Yes	0.509	0.190–1.363	
**Omentectomy**			0.563
No	1		
Yes	0.775	0.326–1.839	
**Decade**			0.717
1st	1		
2nd	1.161	0.517–2.611	

## Data Availability

The data presented in this study are available on request from the corresponding author. The data are not publicly available due to patients’ privacy restrictions.

## Supplementary Materials

The following are available online at www.mdpi.com/article/10.3390/curroncol28060410/s1, Figure S1: Overall survival difference between mixed and pure USCs.
