# Supplementary figures and images for: What Has Changed in the Management of Uterine Serous Carcinomas? Two Decades of Experience
Source: Curr Oncol. 2021 Nov 20;28(6):4862–73. doi: 10.3390/curroncol28060410 (PMC8628752; doi:10.3390/curroncol28060410)

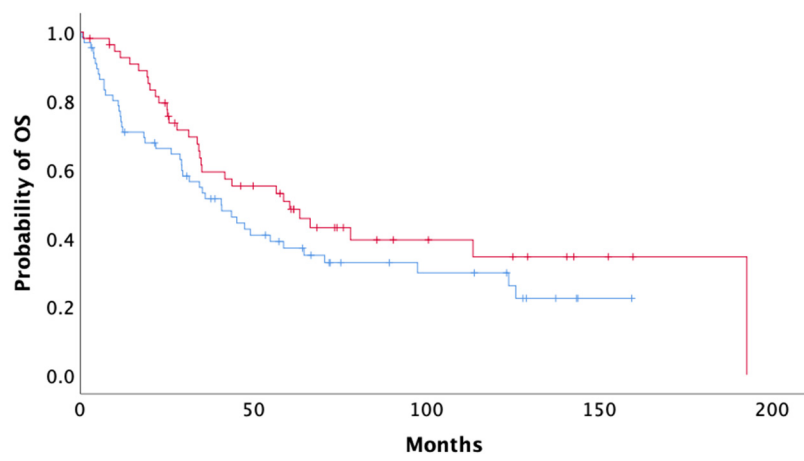

**Figure S1.** Overall survival difference between mixed and pure USCs.

Supplement: Supplementary file 1 [file curroncol-28-00410-s001.zip › curroncol-1388810-supplementary.pdf]
